# Lenalidomide in Multiple Myeloma: Review of Resistance Mechanisms, Current Treatment Strategies and Future Perspectives

**DOI:** 10.3390/cancers15030963

**Published:** 2023-02-02

**Authors:** Piotr Kulig, Sławomir Milczarek, Estera Bakinowska, Laura Szalewska, Bartłomiej Baumert, Bogusław Machaliński

**Affiliations:** 1 Department of General Pathology, Pomeranian Medical University, 70-111 Szczecin, Poland; 2 Department of Hematology and Transplantology, Pomeranian Medical University, 71-252 Szczecin, Poland

**Keywords:** lenalidomide, resistance, LEN-resistant, multiple myeloma, immunomodulatory drug, IMiDs

## Abstract

**Simple Summary:**

Multiple myeloma (MM) is the second most common hematological malignancy. Initially, prognosis for MM patients was poor. However, due to the development of novel treatment regimens, their clinical outcomes have significantly improved. One of the milestones in the treatment of MM was the implementation of immunomodulatory drugs (IMiDs). Lenalidomide (LEN) is probably the most commonly used IMiD worldwide. However, despite its potent anti-MM activity, the vast majority of patients become LEN-resistant. In this review, we focus on LEN-resistance mechanisms as well as on strategies for the treatment of LEN-refractory disease.

**Abstract:**

Multiple myeloma (MM) is the second most common hematologic malignancy, accounting for approximately 1% of all cancers. Despite the initial poor prognosis for MM patients, their life expectancy has improved significantly with the development of novel agents. Immunomodulatory drugs (IMiDs) are widely used in MM therapy. Their implementation has been a milestone in improving the clinical outcomes of patients. The first molecule belonging to the IMiDs was thalidomide. Subsequently, its novel derivatives, lenalidomide (LEN) and pomalidomide (POM), were implemented. Almost all MM patients are exposed to LEN, which is the most commonly used IMiD. Despite the potent anti-MM activity of LEN, some patients eventually relapse and become LEN-resistant. Drug resistance is one of the greatest challenges of modern oncology and has become the main cause of cancer treatment failures. The number of patients receiving LEN is increasing, hence the problem of LEN resistance has become a great obstacle for hematologists worldwide. In this review, we intended to shed more light on the pathophysiology of LEN resistance in MM, with particular emphasis on the molecular background. Moreover, we have briefly summarized strategies to overcome LEN resistance and we have outlined future directions.

## 1. Introduction

Multiple myeloma (MM) is a relatively rare malignancy, accounting for approximately 10% of hematologic neoplasms and 1% of all cancers [1]. Despite being a relatively rare condition, it is the second most common hematologic malignancy [2], making it a major clinical challenge faced by hematologists. The symptomatology of MM is wide and affects numerous organs and systems, significantly hindering normal daily activity and substantially decreasing the quality of patients’ lives. Initially, the life expectancy for MM patients was short. Over time, mainly due to the implementation of novel treatment protocols, the prognosis has improved significantly [3]. There were several milestones in MM management. One of the first breakthroughs in MM therapy was the rediscovery of thalidomide (THAL)—an infamous antiemetic and sedative drug formerly recommended for pregnant women, which was withdrawn due to its teratogenicity [4]. Interestingly, its other properties turned out to be beneficial in MM. Another molecule that significantly improved the prognosis of MM patients was the proteasome inhibitor—bortezomib (BTZ) [5]. The combination of THAL, BTZ and dexamethasone (DXM) is still frequently used as an induction therapy in patients eligible for autologous stem cell transplantation (AHSCT) [6]. Despite its high clinical efficacy, THAL exhibits several adverse drug reactions (ADRs), predominantly peripheral neuropathy (PN), often leading to its discontinuation [7]. Since chemotherapy regimens containing THAL are associated with ADRs, there was an urgent need to develop new derivatives that would maintain a strong therapeutic potential and, at the same time, cause fewer complications. Preclinical studies and subsequent clinical trials resulted in the development and implementation of new immunomodulatory drugs—lenalidomide (LEN) and pomalidomide (POM). Both of them could be incorporated into multiple treatment regimens.

Several mechanisms are postulated to contribute to drug resistance in MM. For instance, it was demonstrated that multidrug resistance-1 (MDR1)/P-glycoprotein (Pgp), an ATP-dependent membrane transporter, promotes the efficient efflux of carfilzomib (CFZ) and doxorubicin in MM [8]. Moreover, the induced expression of proteins such as Pgp is thought to be one of the crucial causes of drug resistance in MM, limiting therapeutic solutions [9]. As mentioned above, novel drugs and treatment protocols have improved the prognosis of MM patients. Nevertheless, over time, there has been a growing subset of patients for whom hitherto effective treatment has lost its clinical efficacy. Drug resistance is a significant challenge for physicians worldwide. Although MM is a disease affecting mainly the elderly, due to the constant increase in life expectancy of the general population, the number of affected patients will definitely increase. Therefore, one may presume that the problem of drug resistance in MM will substantially expand as well. 

Drug resistance is one of the greatest challenges of modern oncology and has become the main reason for treatment failure in cancer. It is estimated that approximately 90% of cancer-related deaths occur as a consequence of drug resistance [10]. Resistance mechanisms can be categorized into intrinsic or extrinsic classes. Intrinsic mechanisms are based on cellular properties, while extrinsic resistance develops during cancer treatment despite primary sensitivity to applied agents [11]. Cellular resistance appears to be mediated by promoted efflux, reduced influx or induction of apoptosis [12]. The activation of alternative signaling pathways and elevated expression of therapeutic targets contribute to extrinsic drug resistance [13]. Anticancer agents eliminate malignant cells. However, at the same time, they exert tremendous environmental pressure on them, eventually leading to the selection of resistant clones. The aim of this review is to shed more light on the pathophysiology of LEN resistance in MM, with particular emphasis on the molecular background, as well as current and possible future strategies for overcoming LEN resistance. Moreover, we have briefly summarized current approaches to the treatment of LEN-resistant MM as well as outlined the future directions.

## 2. Characteristics of Lenalidomide

### 2.1. Overall Characteristic of Lenalidomide

LEN is an immunomodulatory derivative of THAL with more potent anti-MM activity [14,15]. Its toxicity profile is similar to THAL. Nevertheless, typical adverse effects such as somnolence, constipation and PN are less prominent. It is important to notice that significant adverse effect of LEN is its potential to induce cytopenias. These are particularly exemplified by neutropenia and thrombocytopenia, [15] while anemia is noted less frequently [16]. Additionally, in some individuals treated with LEN, dizziness, fatigue and rush were noted. Like its predecessor, LEN was proved to be teratogenic [17]. In addition, both THAL [18] and LEN [19] can induce thrombosis. LEN was established to be effective in patients with newly diagnosed as well as relapsing and refractory MM, initially alone [20] and thereafter in combination with other agents [21,22,23]. LEN is administered orally and subsequently rapidly absorbed. It was demonstrated that the drug is not a substrate for CYP450 nor does it undergo conjugative metabolism. Nevertheless, it undergoes non-enzymatic hydrolysis in hepatocytes as well as in plasma at physiological pH. The vast majority of the drug is excreted unchanged by the kidneys. Therefore, the dosage should be adjusted according to renal function [24]. The maximum tolerated dose was established as 25 mg/day [15]. 

**Figure 1:**
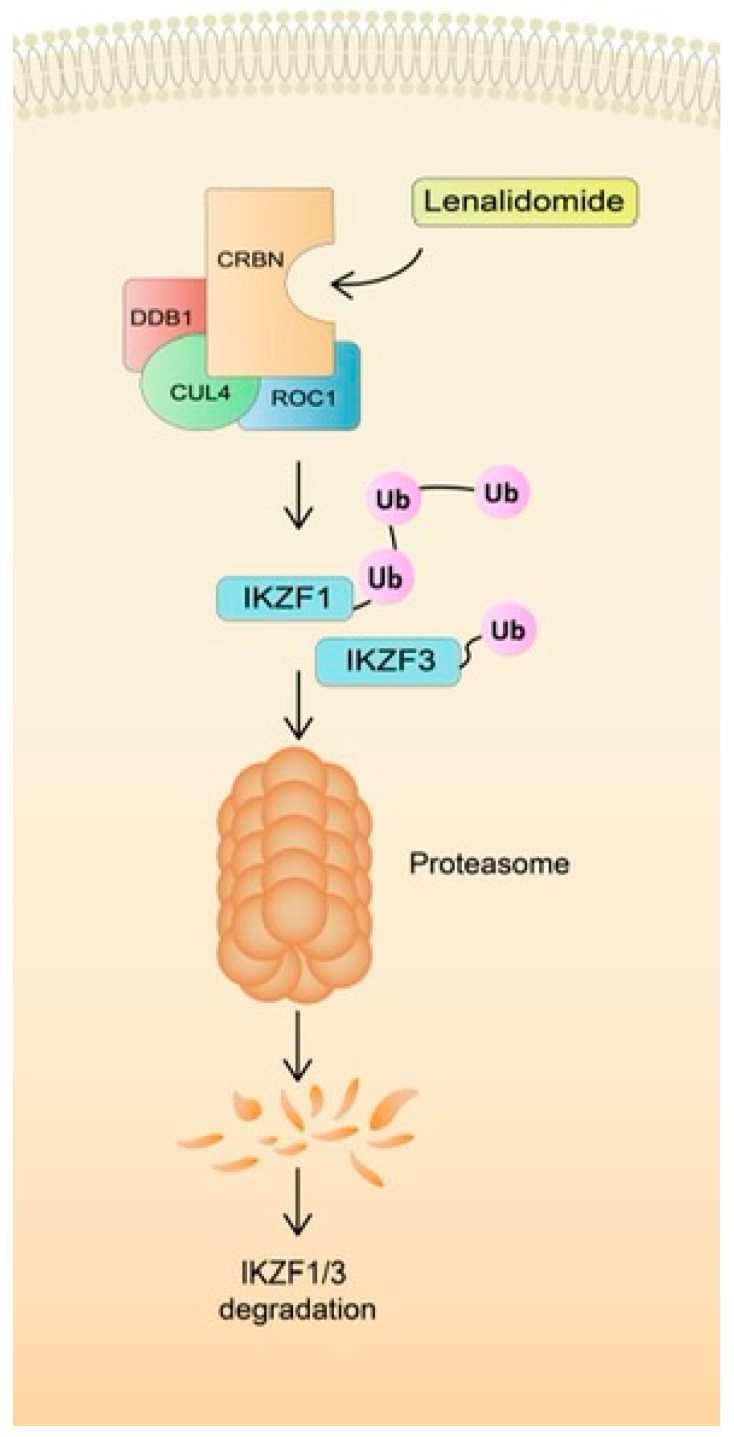
The mechanism of action of lenalidomide.

Despite the potent anti-MM activity resulting in high clinical efficacy, the exact mechanism of action has not yet been determined. The central target molecule for LEN is cereblon (CRBN). CRBN interacts with DNA binding protein 1 (DDB1) and together with cullin-4A (CUL4) and regulator of cullins 1 (ROC1) to form the CRL4 E3 ubiquitin ligase complex (CRL4), which triggers an increase in ubiquitination and subsequent degradation of the two transcription factors Ikaros (IKZF1) and Aiolos (IKZF3) (Figure 1) [25]. IKZF1 and IKZF3 are essential transcription factors for malignant plasma cells. Furthermore, it was established that a single IKZF3 amino acid substitution, knockdown or mutation in CRBN conferred resistance to LEN-induced degradation and rescued LEN-induced inhibition of cell growth. This finding confirms the crucial role of these proteins in the LEN mechanism of action [26]. Multiple other pleiotropic effects result from triggering various downstream mechanisms [27]. Briefly, it was established that LEN induces cell cycle arrest and apoptosis [28]. Moreover, it stimulates T and NK cells by upregulating IL-2 and INF-γ [29]. The anti-angiogenic properties of LEN were also established [30]. This pharmacological effect is particularly important as angiogenesis is a hallmark of MM progression [31]. In addition to the aforementioned well-established mechanisms, LEN induces oxidative stress, which mediates LEN cytotoxicity. Sebastian et al. established that LEN reduces the potential of MM cells to decay H2O2 by intracellular peroxidase. Malignant plasma cells with a decreased ability to decompose hydrogen peroxide were more prone to LEN-induced free radicals accumulation and the resultant cytotoxicity. In addition, CRBN-dependent degradation of Ikaros and Aiolos was mediated by H2O2-related free radical injury [32]. The study conducted by Jiang and team further strengthens the hypothesis of LEN-induced oxidative stress in MM cells and its associated cytotoxicity. Moreover, they determined that agents that exhibit the proclivity to increase the intracellular generation of reactive oxygen species, such as chidamide, may act synergistically with LEN and enhance its anti-MM effect [33]. Taken together, anti-MM properties of LEN are ascribed to various direct and indirect mechanisms that have been thoroughly reviewed elsewhere [34,35,36,37].

### 2.2. Chemical Structure of IMiDs 

**Figure 2:**
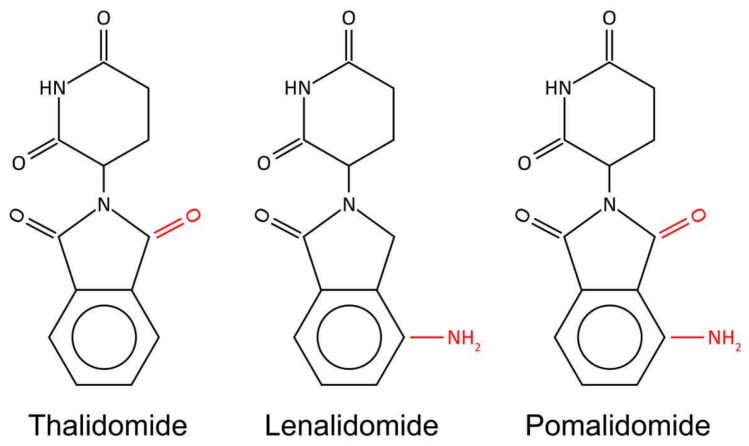
Chemical structure of IMiDs. All three molecules share phthalimide and glutarimide ring and differ only in a carboxyl and amino group.

IMiDs are derivatives of THAL. Therefore, they exhibit a similar mechanism of action, i.e., binding to CRBN elicits their downstream activity, overlapping toxicity profile, as well as shares some adverse reactions. In addition, all molecules have a similar chemical structure. However, there are subtle but important differences between these three agents. All three representatives of IMiDs have a mutual phthalimide and glutarimide carbon skeleton and are distinguished only by the side chain; more precisely, by the carboxyl and amino groups (Figure 2) [25]. 

## 3. Resistance to Lenalidomide

**Figure 3:**
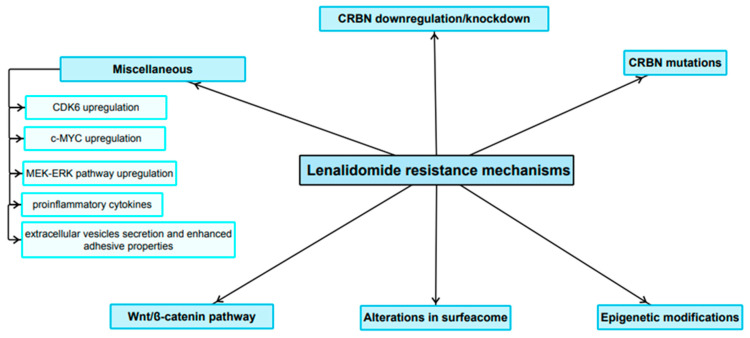
Main LEN-resistance mechanisms.

LEN was proved to be a highly effective anti-MM agent. Nevertheless, over time, MM cells exposed to the drug can evade its target pathways and become resistant to therapy. As mentioned above, the LEN mechanism of action is complex and not entirely elucidated. In addition, it exhibits numerous pleiotropic effects. It can therefore be hypothesized that the molecular mechanisms developed in order to select a resistant clone are at least as complex as the mechanism of action itself. The resistance mechanisms are summarized in Figure 3.

### 3.1. Role of CRBN Suppression in the Development of LEN Resistance

CRBN is the main target molecule for LEN, and triggered downstream mechanisms elicit the vast majority of the pharmacological effects and are responsible for its anti-MM properties [38]. Therefore, it can be hypothesized that multiple acquired abnormalities in CRBN-related pathways in malignant plasma cells result in decreased LEN sensitivity or development of drug resistance. One of the hallmarks of LEN resistance is CRBN loss or downregulation. In order to investigate downstream mechanisms elicited by CRBN suppression and their role in mediating LEN resistance, an analysis was conducted to search for genomic abnormalities and gene expression differences between LEN-resistant and LEN-sensitive cells. The investigation revealed an association of tetraploidy with LEN resistance, and the karyotype was further characterized by the loss of one additional copy of 3p (containing the CRBN locus). Furthermore, CRBN was among the top 10 genes underexpressed in LEN-resistant cells. The lack of CRBN expression was associated with complete resistance to LEN. Similar findings were found in vivo. Myeloma patients with LEN resistance demonstrated a reduction in CRBN expression levels. The study concluded that CRBN is essential for LEN activity, and that low levels of CRBN correlate with poor drug response. Moreover, CRBN has been shown to be a pivotal molecule for the development of LEN resistance [39] The results of another study by Franssen and colleagues revealed similar phenomena. They observed a decrease in CRBN protein levels, both nuclear and cytoplasmic, at the time patients developed LEN-refractory disease compared to the time of diagnosis [40]. Gooding and co-workers investigated alternative genomic associations of the resistance to the immunomodulatory agents implementing whole-genome sequencing (WGS) datasets. They analyzed samples obtained at given timepoints: newly diagnosed, LEN-refractory and LEN-then-POM-refractory states. They identified a chromosome locus 2q37 for which the copy loss significantly enriched among approximately 5% of newly diagnosed, 10% of LEN-resistant and 16.4% POM-resistant MM. Two genes, named COPS7B and COPS8 (both members of the COP9 signalosome), are encoded in this region and are required for CRBN stability. Their partial loss leads to a partial loss of CRBN, which may eventually mitigate LEN/POM efficacy. More precisely, the COP9 signalosome is vital for the perpetuation of the activity CUL4-DDB1-CRBN E3 complex. The study concluded that this region could be a novel IMiD resistance marker with clinical utility [41]. Another study conducted by Liu and co-workers explored the mechanisms underlying the sensitivity of MM cells to IMiDs (including LEN) using CRISPR-Cas9 genome-wide screening. The scientist re-emphasized the role of the signalosome complex and demonstrated a novel molecular mechanism regulating the sensitivity of MM cells to IMiDs. The results revealed that CSN9 signalosome complex regulates IMiD sensitivity by modulating CRBN expression. Briefly, the CSN9 signalosome complex diminishes the SCF^Fbxo7^ E3 ligase-related decay of CRBN. On the other hand, the absence of CSN9 signalosome activity induces the SCF^Fbxo7^ complex and promotes CRBN degradation and mediates resistance to immunomodulatory drugs including LEN [42]. Similar observations regarding the relationship between CRBN downregulation and acquired resistance to LEN have been provided by other studies [43,44]. The utility of baseline CRBN expression as a potential biomarker for responsiveness to LEN is still a subject of an ongoing debate since studies provide contrary evidence. On the one hand, CRBN expression was found to positively correlate with response to LEN [45]. Nonetheless, there are authors whose research concluded otherwise and revealed no predictive value of CRBN expression or protein level for LEN sensitivity [43,44]. As stated above, CRBN is a key molecular target for LEN and alterations in its expression, as well as in downstream signaling, contribute to the development of LEN resistance. Therefore, targeting CRBN in a different way than IMiDs do seems to be plausible research direction. For instance, Zou and team demonstrated that a molecule named BTX306 interferes with CRBN yet exhibits different downstream effects than IMiDs. While IMiDs more significantly promoted the decay of IKZF1 and IKZF3 in MM cells, BTX306 induced greater decrease in concentration of the following molecules on a protein level according to the Western Blot analysis: GSPT1, eRF1, CK1α, MCL-1 and c-MYC. Interestingly, BTX306 exerted anti-MM activity in LEN-resistant lines and had the ability to overcome resistance to BTZ [46]. 

### 3.2. Role of Mutations in CRBN and Genes Encoding Related Downstream Proteins in LEN Resistance

On top of CRBN downregulation, mutations in the CRBN itself or the CRBN pathway appear to mediate LEN resistance. Kortüm and colleagues conducted a study where they performed targeted sequencing to screen 50 multidrug refractory MM patients. Their analysis showed that mutations in the CRBN or IMiD binding site of CRBN appeared to induce LEN resistance. In addition, the longitudinal evaluation of three individuals with CRBN mutations at the onset of IMiD resistance confirmed that these mutations were not detectable before, when they had been sensitive to either LEN or POM [47]. Gooding et al. conducted a study using WGS data from 455 individuals and RNA sequencing (RNASeq) data from 655 subjects, including newly diagnosed MM, LEN-resistant and POM-resistant subpopulations. Their study depicted that CRBN changes at a gene level could be detected in almost 30% of RRMM patients treated with either LEN or POM, rendering it the single most significant contributor conferring resistance in a clinical setting. Moreover, it should be emphasized that overall CRBN expression was lower in IMiD-resistant states [48]. 

Barrio et al. demonstrated that on top of mutations in a gene encoding CRBN at the LEN/POM binding site, other point mutations appeared to play a role in the pathogenesis of LEN/POM resistance in the following manner. They either hindered interactions with other molecules of the complex, or those mutations could destabilize the complex’s effect exerted on its molecular targets. As well as CRBN mutations, point mutations in the genes encoding the CRL4 ligase complex are also involved in the pathogenesis of LEN resistance. Only IKZF1/3 mutations affecting the lenalidomide/cereblon binding site confer LEN-resistance. While alterations in Aiolos, which were possible to detect at the time of diagnosis, appear to contribute the pathogenesis of MM itself rather than to the clinical resistance induced by the long-lasting exposure to the drugs [49]. In an in vitro study conducted on MM cell lines by Zhu et al., a Western blot analysis showed that Ikaros levels were optimal in all LEN-sensitive MM cell lineages that were analyzed. However, levels of the protein were significantly lower in three of the five LEN-refractory cell lineages which were investigated. In the next step, in order to further investigate these phenomena, they implemented flow cytometry analysis to explore IKZF1 expression itself and its alterations after IMiD therapy. The obtained results were coherent with the proteomic analysis. The study’s conclusions implied that mutations in genes encoding proteins of the CRBN-IKZF-IRF4 pathway may eventually elucidate the occurrence of IMiD resistance in more MM individuals than previously thought [50]. 

Tagging a protein with ubiquitin requires three groups of enzymes which are known as E1, E2 and E3. They act cooperatively to ubiquitinate the target protein. LEN interacts with E3 and CRBN forming the CRL4 E3 ligase complex. Nevertheless, Lu et al. demonstrated, using the CRISPR-Cas9 screening approach, that one of the E2 enzymes’, UBE2G1, protein downregulation, gene deletion or mutation confers resistance to both LEN and POM in human myeloma cell lines and may lead to reduced CRL4 CRBN activity. Nonetheless, these MM cells remained sensitive to the more potent IKZF1/3 degrader CC-220. In conclusion, it will be of fundamental importance to investigate whether loss of UBE2G1 activity is associated with clinical resistance in patients not responding to LEN. Moreover, the results imply that the development of novel IMiDs could be of paramount importance, as UBE2G1-depleted cells remained sensitive to a more potent CRBN modulator such as CC-20 [51]. As mentioned above, the ubiquitylation of target molecules requires E1, E2 and E3 enzymes. However, this reaction might be reversed by a special kind of protein—deubiquitinating enzymes (DUBs) [52]. Recently, Van Nguyen demonstrated that one of the DUBs, USP15, antagonized CRL4^CRBN^-induced ubiquitylation of target proteins, and this intercepted their intracellular degradation in a proteasome. This also applies to the LEN-induced decay of Ikaros and Aiolos. Furthermore, the depletion of USP15 enhances LEN-mediated degradation of IKZF1/3 in refractory MM cell lines [53]. 

### 3.3. Role of Epigenetics in the LEN Resistance Development

In addition to the abovementioned mechanisms, it can be hypothesized that epigenetic alterations play a role in the development of LEN resistance. Dimopoulos et al. investigated the mechanisms of LEN resistance using an in vitro model. Their results revealed that LEN-resistant MM cells showed downregulation of CRBN. they then investigated if epigenetic alterations contribute to the development of LEN resistance and CRBN downregulation. Interestingly, they observed that the promoter region of CRBN was not silenced and that the CRBN expression was not under the influence of epigenetic alterations, such as DNA methylation, but possibly by other cis or trans regulatory mechanisms. However, methylation changes did play a role. They established that IMiD-resistance was associated with thorough epigenetic modifications which included changes in chromatin accessibility and DNA methylation. Nevertheless, it was also highlighted that reprogramming did not directly interact with the main components of CRBN-related pathway. Moreover, sensitivity to LEN can be restored with a combination of 5-azacytidine and EPZ-6438 yet in a CRBN-independent manner [54]. On the contrary, Haertle and team came to different conclusions. They examined primary MM tumor samples from 131 patients, including those resistant to LEN according to the International Myeloma Working Group (IMWG). It is vital to emphasize that CRBN has two regulatory regions, a 5′ promoter and located downstream active intronic enhancer. The obtained results revealed DNA hypermethylation in the abovementioned enhancer which were detected samples derived form IMiD-resistant subjects. In addition, methylation was associated with lower CRBN expression. Collectively, these results strongly imply the presence of not currently revealed epigenetic regulation of CRBN which appears to affect the IMiD-based therapy. Furthermore, in the progression free survival (PFS) analysis, newly diagnosed multiple myeloma (NDMM) individuals who underwent IMiD-based treatment regimen, and in whom a hypermethylation of CRBN enhancer was detected, had an inferior PFS [55].

In addition to direct changes in DNA methylation, there are other epigenetic mechanisms contributing to the development of LEN resistance. However, some of them may also be associated with methylation alterations. Of particular relevance are non-coding RNAs that mediate the loss of LEN sensitivity in a variety of ways. Jakobsen et al. investigated the role of circular RNAs (circRNAs) in the mechanisms underlying the development of resistance to IMiDs, including LEN, in an in vitro model. Most notably, they established that genome-wide circRNA expression represented a good response to IMiD, and alterations in its patterns occurred when the IMiDs-refractory state was achieved. It was determined that ciRS-7 was the most depleted circRNA in LEN-refractory MM cell line. The loss of ciRS-7 was evidently associated with the enhanced methylation of the CpG island in the promoter region of its own gene, named LINC00632. The expression level of both molecules, LINC00632 and ciRS-7, was restored to some degree by the treatment of a combination of an EZH2 inhibitor (EPZ-6438) and 5-azacytidine (DNA methyltransferase inhibitor). Interestingly, after such exposure, cells regained the sensitivity. However, a subsequent analysis revealed that ciRS-7 did not appear to be directly involved in the pathogenesis of LEN-resistance development. Nevertheless, alterations in genome-wide circRNA expression patterns are associated with acquired resistance to IMiDs and should be further investigated [56]. 

Another in vitro study by Caracciolo and co-workers yielded interesting results. miR-22 has been found to be associated with LEN sensitivity. Low miR-22 levels were bound to LEN resistance and a poor response to LEN. On the contrary, upregulation of miR-22 correlated with drug sensitivity, predominantly through downregulation of MYC. Furthermore, the study depicted that miR-22 potentiates NK-mediated cytotoxicity induced by LEN. Interestingly, miR-22 overexpression restored LEN sensitivity in MM cell lines with a drug-resistant phenotype. Taken together, these data indicate that miR-22-induced downregulation of MYC is able to potentiate both direct LEN- and NK-mediated cytotoxicity in MM cells [57]. 

Despite emerging evidence that epigenetic mechanisms play an important role in the pathogenesis of the development of LEN resistance, little is still known due to the complexity of the interactions involved. It was established that DNA methylation, as well as non-coding RNA such as miRNA and circRNAs, at least partially explain this phenomenon. Further research in this area is of paramount importance due to the potential reversibility of epigenetic alterations and, thus, clinical utility for relapsed and refractory multiple myeloma (RRMM) patients. 

### 3.4. The Expression Pattern of Certain Surface Antigens Associated with Diminished LEN Sensitivity

The expression pattern of particular clusters of differentiation (CD) or other surface antigens appears to be associated with LEN resistance or diminished drug sensitivity. For instance, Kawano and colleagues investigated the expression of CD138 in MM cells and the resultant clinical implications. The obtained results showed that decreased expression of CD138 was associated with the immature phenotype of MM cells and poor prognosis, even in a subpopulation of patients treated with high-dose chemotherapy. Moreover, it was bound with refractoriness to LEN. The underlying mechanisms were complex and not entirely elucidated. Nonetheless, the authors hypothesized that the observed downregulation of interferon regulatory factor 4 (IRF4) may at least partially explain reduced LEN sensitivity [58]. On the other hand, MM cells overexpressing CD44 exhibited enhanced adhesive properties to bone marrow stromal cells and were resistant to LEN. Furthermore, the results revealed that the role of Wnt/β-catenin axis is of paramount importance in mediating LEN resistance in MM cells overexpressing CD44. In addition, CD44 blockade with monoclonal antibodies, free hyaluronan or CD44 knockdown reduced adhesion and restored LEN sensitivity. Similar effects were elicited by all-trans retinoic acid, which depleted total β-catenin and reduced both cell surface and total CD44 levels. Moreover, the adhesive properties of LEN-resistant myeloma cells were also diminished. Furthermore, the drug activity in LEN-resistant murine in vivo xenograft model was also greater [59]. 

Glucose-regulated protein (GRP) 78 belongs to the heat shock protein (HSP) 70 family with chaperone activity. Rasche et al. demonstrated that there is a variation in GRP78 expression on the cellular surface starting from monoclonal gammopathy of undetermined significance (MGUS) to advanced relapsed and refractory to BTZ and LEN MM. The more advanced the MM, the greater the expression of GRP78, including LEN-resistant MM cells. Interestingly, the combined treatment of LEN and PAT-SM6 (anti-GRP78 monoclonal antibody) exhibited synergistic effects and restored LEN sensitivity in previously resistant cells. Moreover, a 62-year-old man with triple refractory MM was treated with PAT-SM6, BTZ, and LEN achieved partial remission of both intra- and extramedullary lesions [60]. Ferguson et al. conducted an interesting study by thoroughly exploring and profiling the surface antigens of MM cells under various circumstances. First, they determined that the pattern of their expression on MM cells evolved upon drug exposure. During the investigation of alterations in surfaceome elicited by LEN, their results revealed that the most notable common signature was increased in expression of CD33 and CD45/PTPRC in LEN-resistant cell lines [61]. 

### 3.5. Wnt/β-Catenin Pathway

The Wnt/β-catenin pathway is vital in both embryonic development and in the maintenance of proper function of mature tissues. Once activated, the typical Wnt pathway stabilizes the β-catenin and moves it into the nucleus. This promotes the upregulation of various genes which control multiple cellular pathways and processes including cell proliferation, survival, differentiation and migration [62]. What is particularly interesting with regard to CRBN and IMiD molecular actions, Wnt could mediate the CRBN-dependent degradation of proteins. These substrates include, for instance, casein kinase 1α, which negatively regulates Wnt signaling. Furthermore, CRBN was demonstrated to be a positive regulator of Wnt activity [63]. Therefore, it can be hypothesized that Wnt/β-catenin pathway may be involved in LEN activity, and alterations in this particular signaling pathway may contribute to the development of IMiDs resistance. Indeed, recent evidence suggests that it also contributes to the development of LEN resistance in MM. Bjorklund and co-workers observed that overactivation of Wnt/β-catenin signaling conferred LEN resistance, and conversely, suppression of the pathway resulted in restoration of LEN sensitivity. Interestingly, LEN itself induces β-catenin expression, which may eventually lead to resistance [64]. In addition, as mentioned above, overactivation of the Wnt/β-catenin pathway induced by CD44 overexpression was showed to be an additional mechanism for the development of LEN resistance [59]. 

### 3.6. Miscellaneous 

There are also other mechanisms contributing to the development of LEN resistance that cannot be assigned to the abovementioned categories, but which may be important for future study design. Therefore, we believe that these studies are worth including and briefly summarized in our review.

Cyclin-dependent kinase 6 (CDK6) is a vital protein for the proper regulation of the cell cycle. It was demonstrated by Ng and team that the upregulation of CDK6 decreased the sensitivity of MM cells to LEN, which contributes to the development of resistance. In addition, the study depicted that inhibition or degradation of CDK6 was highly synergistic with immunomodulatory drugs and potentiates its anti-MM effects. It was concluded that CDK6 upregulation is a drug-susceptible target in IMiD-resistant MM [65].

Mori and colleagues demonstrated a novel mechanism for the development of multidrug resistance in MM, including refractoriness to LEN. In the first place, they determined that high MYC expression correlates with low nuclear receptor co-repressors 2 (NCOR2) expression, which belongs to nuclear receptor co-repressors that activate histone deacetylase and alter epigenomic modification. Thereafter, they established a LEN-resistant MM cell line. A subsequent analysis showed that long-term IMiD treatment induced NCOR2 mutation and NCOR2 downregulation. Next, they tested LEN- and POM-resistant cell lines with CPI0203 (bromodomain and extra-terminal domain inhibitor)/ACY1215 (histone deacetylase 6 inhibitor). Both cell lines showed significant resistance to both agents, suggesting that MYC upregulation contributes to multidrug resistance [66].The term SUMOylation is utilized to define a particular post-translational modification which involves the covalent bond of certain proteins named small ubiquitin-like modifier (SUMO) to lysine residue on target molecules. Expression of SUMO E1 was shown to be upregulated in LEN-resistant MM cells. SUMOylation inhibition restored LEN sensitivity. Importantly, it was associated with MYC and IRF4 downregulation. On one hand, this study revealed a novel mechanism mediating LEN resistance, and on the other, it further highlighted the role of MYC upregulation [67]. Moreover, another study depicted that role of increase in c-Myc expression at the onset of LEN-resistant disease [40]. 

Ocio et al. conducted a very interesting in vivo study where they established a xenograft model resistant to LEN plus DXM and POM plus DXM. First, the results of the study showed no cross-resistance between IMiDs. Subsequently, it was demonstrated that mice, which had become refractory first to one treatment regimen and subsequently to the next, were re-treated with the initial regimen (either RD or PD) after a wash-out period without treatment. Interestingly, the tumors again responded to treatments to which they had initially developed resistance. In conclusion, the results revealed an upregulation of the MEK/ERK pathway in acquired LEN and POM resistance. Interestingly, the addition of a MEK inhibitor reversed IMiD resistance in both in vitro and in vivo models [68]. In another study, genome-wide CRISPR-Cas9 knockout (KO) screening was carried out to identify genes and/or pathways mediating IMiD sensitivity. The tumor necrosis factor (TNF) receptor-associated factor 2 (TRAF2) gene was identified as a major modulator of LEN sensitivity. Indeed, TRAF KO resulted in acquired resistance to LEN in MM cells. Subsequently, underlying mechanisms were investigated. TRAF2 KO induces activation of non-canonical NF-κB and MEK-ERK pathways. More precisely, the non-canonical NF-κB pathway appears to regulate MEK-ERK activity. Interestingly, drug resistance is not specific to IMiDs (cells became resistant to melphalan and DXM) and independent of the ubiquitin-proteasome pathway. Further analysis showed that within MM bone marrow (BM) stromal cell supernatants (MM cells were co-cultured with bone marrow stromal cells), TNF-α induces proteasomal degradation of TRAF2, non-canonical NF-κB and downstream ERK signaling in MM cells, whereas interleukin-6 directly triggers ERK activation. The study delineated a novel, CRBN-independent mechanism of IMiD resistance in the BM milieu [69].

Wang and co-workers demonstrated that downregulation of the chemokine CCL20 was associated with LEN resistance, and the addition of CCL20 restored LEN sensitivity in both in vitro and xenograft models [70]. Another study, which covered the role of cytokines in the development of LEN resistance, was conducted by Wu and colleagues. They highlighted the role of the IL-6-heme oxygenase-1 (HO-1) axis. First, they determined that both serum IL-6 levels and IL-6 mRNA expression levels within cells correlated with HO-1 expression in the bone marrow of CD138^+^ cells from MM individuals, and that exogenous IL-6 upregulated HO-1 in malignant plasma cells. IL-6-induced high expression of HO-1 allowed MM cells to resist LEN. This was hypothetically attributed to the IL-6-mediated activation of the JAK/STAT3 pathway. However, diminishing the expression of IL-6 mediated by HO-1 re-sensitized MM cells to LEN [71]. Colombo demonstrated that the inhibition of Notch1 and 2 signaling by targeting their ligands named Jagged1 and 2 can re-sensitize MM cells to LEN, BTZ and melphalan, implying that this pathway may mediate multidrug resistance. Further investigation revealed that chemokine axis CXCR4/SDF1α might be involved [72]. 

Yamamoto et al. demonstrated another molecular and cellular mechanisms of LEN resistance in MM. In comparison to LEN-sensitive MM cells, cells that exhibited the resistant phenotype were characterized by the increased secretion of extracellular vesicles (EV) and elevated adherence abilities. A whole transcriptome analysis revealed that the Sortilin 1 (SORT1) and Lysosomal Associated Membrane Protein 2 (LAMP2) genes were core regulatory genes of EV secretion. In addition, their knockout resulted in lowering EV secretion and the loss of adhesive properties of resistant cells, eventually leading to greater LEN sensitivity [73]. Similarly, Hattori and co-workers showed that long-term exposure to LEN can result in cell adhesion-mediated drug resistance. MM cells exhibiting the resistant phenotype were characterized by the overexpression of integrin β5 and β7 [74]. 

CCAAT/enhancer-binding protein-β (C/EBPβ) is one of crucial regulators of growth and differentiation of B cells. IMiD compounds, LEN and POM downregulate eIF4E, which prevents the translation of C/EBPβ and, as a result, inhibits IRF4 transcription. This in turn decreases the expression of the network of IRF4-dependent transcription factors, eventually leading to the inhibition of MM proliferation. On the other hand, the overexpression of C/EBPβ mediates IMiD resistance, emphasizing the role of C/EBPβ in promoting resistance to LEN and POM [75].

## 4. Current Therapeutic Strategies and Future Perspectives

### 4.1. Restoration or Modulation of CRBN Signaling in Resynthesis of MM Cells to IMiDs

It appears that low CRBN expression, mutations in CRBN and in genes encoding downstream proteins collectively contribute to the development of LEN resistance. Nevertheless, alterations in CRBN itself or its downregulation seem to be the principal component. This was demonstrated in a study by Zhu and colleagues, which depicted that CRBN depletion and mutations should be considered as the most prevalent mediators of acquired resistance to IMiDs in MM. Furthermore, they also described a new mechanism of resistance driven by a proinflammatory cytokine—IL-6 and the activation of STAT3 [76]. 

Therefore, modulation or restoration of CRBN signaling appears to be a viable research direction with potential clinical implications. Hansen and colleagues conducted an interesting in vitro study where they investigated possible options to overcome LEN resistance. The obtained results showed that LEN sensitivity can be restored despite low intracellular LEN levels, as in the H929 R10-1 MM cell line. They described a CRBN E3 ligase modulator named CC-92480, a molecule acting as though it was a molecular glue, facilitating the interplay between IKZF1 and CRBN and promoting targeted docking to the CRL4-CRBN E3 complex. CC-92480-dependent attachment of IKZF1/3 to CRBN results in polyubiquitination and eventually proteasome-mediated decay of a protein, which results in the subsequent apoptosis of LEN-resistant MM cells [77]. 

### 4.2. Lack of Cross-Resistance within the Immunomodulatory Drug Class 

**Figure 4:**
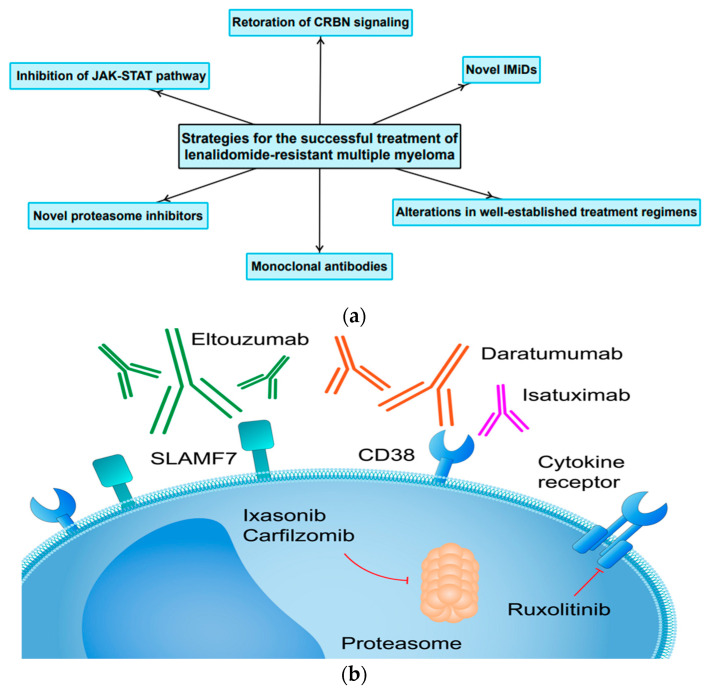
(**a**) The most important treatment strategies and future perspectives for the successful treatment LEN-resistant MM. (**b**) Molecular targets for novel agents successfully implemented in the treatment of RRMM.

LEN is a potent anti-MM drug. Nevertheless, despite its high clinical efficacy, there is a patient population that has become resistant to LEN. Therefore, the development of novel molecules, the implementation of different treatment regimens, as well as basic research in this field are of great importance. Figure 4 depicts the most important therapeutic strategies for the successful treatment of LEN-resistant MM as well as future perspectives.

There is ample evidence that cross-resistance within immunomodulatory drug class does not exist. In the first place, LEN was proved to be effective in THAL-resistant patients [78]. Subsequently, the efficacy of POM (in combination with DXM), a novel immunomodulatory agent and a derivative of LEN, was established in a preclinical model in both LEN-sensitive and LEN-resistant MM cell lines, demonstrating potent anti-proliferative and pro-apoptotic activity [79]. Similar results were observed in a murine xenograft model [68]. Moreover, several clinical trials have been conducted proving the effect of POM against MM in relapsed and refractory LEN-resistant MM. Initially, it was established in 1/2 phase study that the combination of POM, BTZ and DXM (PVd) was well tolerated and highly active in patients with RRMM who were refractory to LEN and had been previously exposed to BTZ [80]. Another 1/2 phase study demonstrated the efficacy and tolerability of the PVd regimen in LEN-resistant population of patients. Thereafter, the results of a phase 3, randomized, open-label trial named OPTIMISMM further supported the use of PVd regimen as a treatment option for patients with relapsed or refractory MM who had previously received LEN [81]. Moreover, a sub-analysis of the aforementioned clinical trial demonstrated the efficacy of PVd regimen in patients who developed resistance to LEN as a very next line of therapy. Most importantly, these findings indicate that there is no need to replace the immunomodulatory agent with another class of drugs after LEN treatment fails [82]. Patients enrolled to clinical trials belong to highly selected populations. Therefore, there is a possibility of a discrepancies between “the real life” results and those presented after the completion of clinical trials. According to a retrospective study conducted by the Polish Myeloma Group, POM was proved to be an active drug in RRMM, which is supported by the results of published clinical trials. The study revealed the following results with regard to overall response rate (ORR), PFS and overall survival (OS): ORR was established at a level of 39.1%, while the median PFS and OS were demonstrated to be 10.0 and 14.0 months, respectively. Importantly, previous treatment with IMiDs, BTZ or stem cell transplant did not influence PFS and OS [83]. 

### 4.3. Unconventional Changes to Already Existing Treatment Regimens for LEN-Resistant MM

As well as novel agents which are currently being investigated in clinical trials, the development of alternative treatment regimens consisting of already existing drugs should be considered and explored. Unconventional modification can lead to a good clinical response. Furthermore, in the vast majority of cases it is an efficient and cost-effective option that can be quickly implemented. Ghosh at al. conducted a retrospective study in which they analyzed 24 patients treated with LEN and DXM (Rd). After confirming disease progression to Rd, clarithromycin was added to Rd treatment regimen which remained unchanged in terms of dosage. The overall beneficial response rate (CR + VGPR + PR + MR − minimal response) was relatively high—45.8% (95%: CI 25.6, 67.2). A survival analysis revealed that the median PFS was demonstrated to be 4 months and 25 months of median OS with the median follow-up of 27.5 months. Of particular interest, high-risk cytogenetics (FISH) had no significant negative impact on PFS, but adversely affected OS, indicating a poor response of these patients to further treatment. The study concluded that the addition of clarithromycin to LEN and DXM could overcome resistance to LEN and DXM and lead to durable clinical responses [84]. 

Kalff et al. conducted a clinical trial where they examined the efficacy of oral azacytidine (AZA) in LEN-resistant MM. They showed that in a group of previously heavily treated LEN-resistant, RRMM patients, meaningful clinical responses were achieved without significant toxicity when treated with AZA in combination with the Rd regimen. Interestingly, proteomic analysis revealed that higher CRBN protein expression in MM cells before treatment correlated with superior outcomes [85]. However, the results of a subsequent study by Khouri et al. were less optimistic. They demonstrated tolerability of twice-weekly subcutaneous AZA 50 mg/m^2^ with Rd in RRMM and implied that AZA might be putatively beneficial in an attempt to face the LEN/POM refractoriness, presumably via activating differentiation pathways. The relatively low response rates and the relation of the satisfactory clinical outcome with low plasma levels of the AZA inactivating enzyme named CDA imply that the AZA-based regimen will require further adjustments. Moreover, a careful patient inclusion criteria may be essential to maximize clinical outcomes [86]. 

Additionally, cyclophosphamide (CTX) has the ability to overcome LEN resistance. Alahmadi and team showed that CTX added to the Rd regimen at the time of diagnosis of disease progression is clinically beneficial. The obtained results revealed that the overall response rate at the level of at least PR (PR, VGPR or CR taken together) was 34%, and clinical benefit (at least stable disease) was observed in the vast majority of enrolled individuals (87%). The median length of therapy with the regimen Rd + CTX was 6.9 months. The median PFS was 6.1 months from the addition of CTX and 24.1 months from the start of Rd [87]. Another alteration of the Rd regimen was demonstrated in a study conducted by Zelis and co-workers. In RRMM patients treated according to the Rd protocol, the following modifications were made: DXM was discontinued, and patients received low-dose oral CTX in combination with prednisone. The study found prednisone treatment to be an effective and well-tolerated regimen in RRMM patients who had previously been exposed to standard Rd therapy [88]. Similarly, the antiretroviral agent nelfinavir has been found to overcome LEN resistance in RRMM when incorporated into Rd regimen [89]. 

The abovementioned alterations and modifications of well-established treatment regimens are not an alternative to novel agents with more potent anti-MM activity. However, they can be considered an easily accessible and cost-effective option in regions and institutions where new anti-MM drugs are, due to various reasons, difficult to obtain. 

### 4.4. The Application of Monoclonal Antibodies and Novel Proteasome Inhibitors in Patients with LEN-Resistant MM

Daratumumab is an anti-CD38 antibody that exhibits anti-MM activity through various mechanisms. The combination of daratumumab (DARA), BTZ and DXM in RRMM was investigated and compared to a regimen consisting of BTZ and DXM solely. The combination tested in the study arm was found to be more effective against RRMM, including the LEN-resistant subset of patients [90]. Another anti-CD38 antibody is Isatuximab (IXA). The ICARIA trial evaluated the addition of IXA to POM and DXM versus POM and DXM. The vast majority of patients enrolled to the study were LEN-resistant. The results showed the superiority of the combination of IXA, POM and DXM compared to the control group. This, in turn, suggests that the addition of a monoclonal antibody enhances the effect of POM [91], which has already been shown to be effective in LEN-resistant MM in the OPTIMISMM trial [81,82]. Elotuzumab (ELO) is a humanized monoclonal antibody targeting signaling lymphocytic activation molecule F7 (SLAMF7). A triplet treatment regimen consisting of ELO, POM and DXM was tested in LEN and proteasome inhibitor-resistant MM. The abovementioned combination was shown to reduce the risk of progression or death compared to the control group (POM plus DXM) [92].

Carfilzomib (CFZ) is a proteasome inhibitor which is a derivative of BTZ. The ENDEAVOUR clinical trial established that the combination of CFZ and DXM is a superior therapeutic option than BTZ and DXM. Importantly, the investigated treatment regimen was found to be effective in LEN-resistant MM [93]. Similarly, ixasonib, a novel oral proteasome inhibitor, has been proven to be a feasible therapeutic option for LEN-resistant patients in combination with either POM and DXM [94] or one of these agents [95]. The CANDOR study evaluated the efficacy and safety of CFZ, DXM, and DARA versus CFZ and DXM in patients with RRMM (including LEN-resistant MM). The results of the study revealed that CFZ, DXM, and DARA (KdD) significantly prolonged PFS versus Kd in patients with RRMM and were associated with a favorable benefit-risk profile [96]. These clinical trials provided promising results. However, as mentioned earlier, patients enrolled to the clinical trials belong to a highly selected population and must fulfill prespecified inclusion criteria. Thus, they often differ from the representative population of patients suffering from the disease under study. Kawaji-Kanayama and colleagues from The Kyoto Clinical Hematology Study Group conducted a real-life study to assess the real-world efficacy and safety of CFZ-based treatments. The study showed that a CFZ-based protocol is a feasible option for patients with RRMM. Nevertheless, resistance to either LEN or BTZ was associated with a worse clinical outcome. Moreover, it has been shown to be an independent unfavorable factor for both PFS and OS. Therefore, there is a need to development a novel therapy based on CFZ, for instance with monoclonal antibodies or other potent anti-MM agents that can fully overcome the clinical resistance to LEN [97].

### 4.5. The Role of JAK-STAT Pathway

Inhibition of the JAK-STAT pathway may be an interesting novel therapeutic approach. This pathway contributes to the development and progression of MM among others due to interaction with BM microenvironment. For instance, macrophages have the ability to differentiate into either M1 (anti-cancer activity and production of inflammatory cytokines) or M2 (stimulating angiogenesis, tumor growth and metastasis). Chen and colleagues conducted an interesting study where they demonstrated in a preclinical model of MM that inhibition of the JAK pathway suppresses M2 polarization. In addition, they showed that ruxolitinib (an inhibitor of the JAK pathway) can overcome LEN resistance [98]. Furthermore, the feasibility of interfering with JAK-STAT pathway was investigated in phase I clinical trials. Ruxolitinib has been found to be an interesting therapeutic option. Simultaneously, it was demonstrated that this agent can overcome resistance to LEN, representing a new and promising therapeutic approach for MM patients [99,100].

## 5. Conclusions

LEN resistance appears to be mediated predominantly through alterations in CRBN-mediated signaling. Nevertheless, other mechanisms also contribute to this phenomenon. Identifying the molecular pathways underlying LEN resistance is of a paramount importance. First of all, because they may emerge in the future as potential therapeutic strategies and become the subject of translational research, and consequently improve patients’ outcomes. Moreover, many new therapeutic strategies are available for the successful treatment of LEN-resistant MM. Of particular importance are monoclonal antibodies and novel derivatives of currently used antiMM agents. At the same time, drugs widely used in other diseases, such as JAK-STAT pathway inhibitors, should be investigated in MM, especially when their mechanism of action may interfere with pathophysiological pathways involved in the development and progression of MM.

## Data Availability

Not applicable.
